# Therapeutic Hints

**Published:** 1895-05

**Authors:** C. M. Boger

**Affiliations:** Parkersburg, W. Va.


### THERAPEUTIC HINTS.

C. M. BOGER, M. D., PARKERSBURG, W. VA.

1. Menses stain indelibly—*Viburnum*, *Medorr.*, Bursa-past., Mag-sul.

Hæmaturia, especially if due to abuse of Quinine—Boracic-acid.

Neuralgic pains which leave the bone sore—HEPAR-S.

In burning pains Kreosote stands next to Arsenicum, and cures frequently after failure of the latter.

Intoxication from the vapor or fumes of gasoline, benzine, etc., or symptoms in workmen handling them, generally require Gelsemium.

Abuse of snuff, resulting in constipation, with or without vertigo—Silicea.

